# Phenotypic and Genotypic Characterization of *Staphylococcus aureus* Isolated from Nasal Samples of Healthy Dairy Goats in Algeria

**DOI:** 10.3390/pathogens13050408

**Published:** 2024-05-15

**Authors:** Yacine Titouche, Madjid Akkou, Allelen Campaña-Burguet, Carmen González-Azcona, Yasmina Djaoui, Donia Mechoub, Abdelhak Fatihi, Pascal Bouchez, Laurence Bouhier, Karim Houali, Yacine Nia, Carmen Torres, Jacques-Antoine Hennekinne

**Affiliations:** 1 Laboratory of Analytical Biochemistry and Biotechnology (LABAB), University Mouloud Mammeri, Tizi Ouzou 15000, Algeria; ymndjaoui@gmail.com (Y.D.); ineesmec@gmail.com (D.M.); houalitizi@yahoo.fr (K.H.); 2 Institute of Veterinary Sciences, University of Saad Dahlab Blida 1, Blida 09000, Algeria; akkoumadj@gmail.com; 3 Area of Biochemistry and Molecular Biology, OneHealth-UR Research Group, University of La Rioja, 26006 Logroño, Spain; allelencampanaburguet@gmail.com (A.C.-B.); carmen.gonzalezaz@unirioja.es (C.G.-A.); carmen.torres@unirioja.es (C.T.); 4 University Paris Est, Anses, Laboratory for Food Safety, F-94700 Maisons-Alfort, France; abdelhak.fatihi@anses.fr (A.F.); pascal.bouchez@anses.fr (P.B.); laurence.bouhier@anses.fr (L.B.); yacine.nia@anses.fr (Y.N.); jacques-antoine.hennekinne@anses.fr (J.-A.H.)

**Keywords:** *Staphylococcus aureus*, nasal carriage, molecular characterization, antimicrobial resistance, MLST

## Abstract

The present study aimed to determine the phenotypic and genotypic characteristics of *S*. *aureus* isolates from the nasal swabs of goats. A total of 232 nasal samples (one per animal) were collected from goats on 13 farms located in two regions of Algeria and were analyzed for the presence of *S*. *aureus*. The detection of virulence factors was carried out using PCR. The antibiotic susceptibility of the recovered isolates was assessed using the disc diffusion method. The biofilm formation ability was assessed by the Congo red agar method and a microtiter plate assay, and the molecular characterization of isolates was carried out by *spa*-typing, and for selected isolates also by multilocus sequence typing (MLST). Overall, 36 out of 232 nasal swabs (15.5%) contained *S*. *aureus*, and 62 isolates were recovered. Regarding the virulence factors, at least one staphylococcal enterotoxin gene was detected in 30 (48.4%) isolates. The gene *tst* encoding the toxic shock syndrome toxin was detected in fifteen isolates (24.2%), but none of the isolates harbored the gene of Panton–Valentine leukocidin (*lukF*/*S*-PV). Nine different *spa*-types were identified, including the detection of a new one (t21230). The recovered isolates were assigned to three clonal complexes, with CC5 (51.8%) being the most common lineage. Two isolates were methicillin-resistant (MRSA) and belonged to ST5 (CC5) and to *spa*-types t450 and t688. Moreover, 27 (43.5%) of the *S*. *aureus* isolates were found to be slime producers in Congo red agar, and all of the recovered isolates could produce biofilms in the microtiter plate assay. Our study showed that the nares of healthy goats could be a reservoir of toxigenic and antibiotic-resistant strains of *S*. *aureus* isolates, including MRSA, which could have implications for public health.

## 1. Introduction

The use of antimicrobial agents in animals for therapeutic or prophylactic purposes, as well as for animal growth promotion, significantly contributes to the development of antimicrobial resistance (AMR), a growing public health threat [1]. Since 2006, the European Union has banned the use of antimicrobial agents as animal growth promoters, as did some other countries; nevertheless, this usage practice is still authorized in about 25% of countries at a global level [2]. Many of the antimicrobials administered to food animals belong to the same family group as those used in human medicine, including penicillins, tetracyclines, cephalosporins, and fluoroquinolones [3]. The increase in antimicrobial usage is correlated with the emergence of AMR in livestock animals [4]. Therefore, farm animals are a significant source of multidrug-resistant bacteria and antimicrobial-resistant determinants [5]. These bacteria include many zoonotic organisms that are frequently resistant to antibiotics, such as *Salmonella*, *Escherichia coli*, and *Staphylococcus aureus*, among others [6,7,8].

In humans and many animal species, *Staphylococcus aureus* is considered to be a major opportunistic pathogen [9]. It can cause a large array of infections, ranging in severity from superficial skin infections to more severe diseases, such as endocarditis, toxic shock syndrome, septicemia, and necrotizing pneumonia, among others [10]. *S*. *aureus* also causes a variety of infections with considerable economic impacts in livestock animals, including cows, sheep, goats, poultry, and rabbits [11]. The most common disease in ruminants is mastitis, which is an inflammation of the udder tissue leading to abnormalities in milk production [12]. *S*. *aureus* colonizes its hosts without impacting their health, as is the case for any type of commensal bacterium [13]. It has been reported to colonize the nares of 30% of humans and practically all domesticated farm animals, including pigs, cattle, poultry, as well as companion animals like cats, dogs, and horses. It has also been found in wild animals [14].

The most challenging characteristic of *S*. *aureus* that has become a global health concern is its capacity to acquire resistance against several antibiotic molecules, including methicillin [15]. Methicillin resistance is conferred by the *mecA* gene, which encodes a modified penicillin-binding protein, PBP2a (or PBP2′), with a low affinity for most β-lactam antibiotics [16,17,18]. The *mecA* gene is part of a large mobile genetic element named staphylococcal cassette chromosome *mec* (SCC*mec*) [19]. In the past, methicillin-resistant *S*. *aureus* (MRSA) has been associated with infections in health-care settings, and these strains have been named hospital-acquired MRSA (HA-MRSA). However, MRSA infections have also been reported outside hospitals in healthy people with no prior exposure to these hospital structures (CA-MRSA) [20]. Recently, livestock-associated MRSA (LA-MRSA), mainly the complex clonal CC398, has been implicated in community infections [21]. LA-MRSA strains differ from HA-MRSA and CA-MRSA in their genomic traits [22]. As reported, livestock can be considered sources of MRSA, which can be transmitted to humans in close contact with farm animals [19], such as farm workers and their family members, veterinarians, and veterinary students [23]. Moreover, the handling or consumption of foods of animal origin contaminated with MRSA, including milk and meat, can also be involved in MRSA transmission through the food production chain [24].

The increased number of reports describing community infections and the emergence of new highly virulent clones highlight the crucial importance of identifying potential reservoirs for newly emergent strains in humans [11]. The aims of this study were to determine the occurrence of *S*. *aureus* in the nasal swabs of healthy dairy goats in two regions of Algeria (Tizi Ouzou and Bouira) and to characterize the recovered isolates both phenotypically and genotypically.

## 2. Materials and Methods

### 2.1. Ethical Approval and Consent to Participate

This study was approved on 4 February 2021 by the internal ethics committee of the University Mouloud Mammeri of Tizi Ouzou, Algeria (Eth-Com/UMMTO/2021/23-Ani). The nasal swab samples were collected from each goat farm under the written informed consent of farm owners, who were informed of the objectives of this work and the sample collection method.

### 2.2. Sample Collection

**Figure 1:**
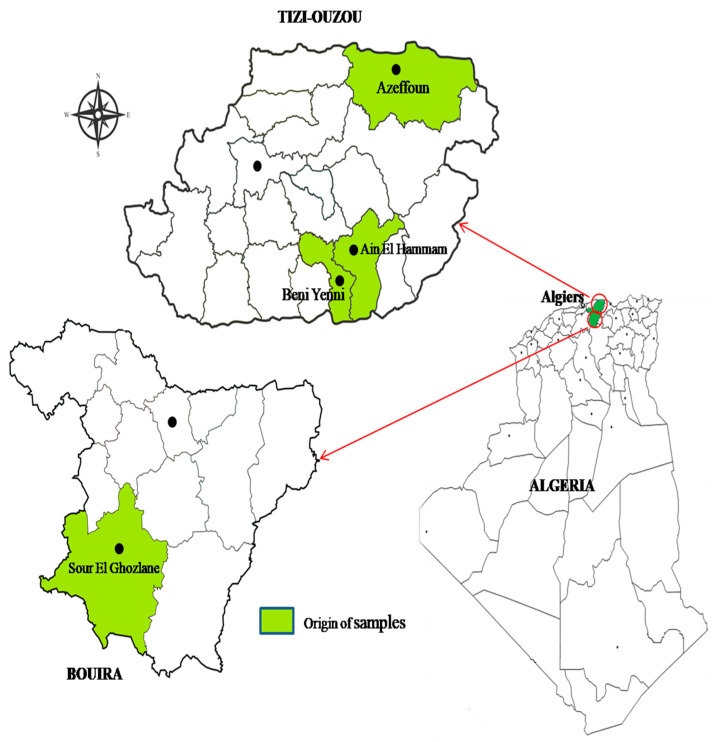
Geographic distribution of the dairy goat farms at Tizi Ouzou and Bouira provinces of Algeria in which the nasal samples were taken to be analyzed in this study.

Between March and June 2021, the nasal swabs of 232 goats (one sample/animal) were collected from 13 dairy goat herds located in different regions of two provinces of Algeria: Tizi Ouzou (Azeffoun, Ain El-Hammam et Beni Yenni) and Bouira (Sol-El-Ghozlane). The region of Tizi Ouzou is located on the central coast of Algeria and is characterized by its mountainous relief. On the other hand, the region of Bouira is located in the center of Algeria and is characterized as plain and partially under the influence of the Sahara (Figure 1). At the time of sampling, none of the screened goats presented apparent clinical symptoms of infection and none of them had received antibiotic treatment. The nasal samples were taken by swabbing both nares of each goat with a sterile cotton swab, after proper cleaning and disinfection of the external nares with cotton soaked with 70% ethyl alcohol. The collected nasal samples were introduced to tubes containing Mueller–Hinton broth with 6.5% NaCl and transported to the laboratory in cooled containers for microbiological analysis.

### 2.3. Isolation of S. aureus

Firstly, an enrichment step was performed through inoculation of the collected nasal samples in Mueller–Hinton broth (Conda Pronadisa, Madrid, Spain) supplemented with 6.5% NaCl (*v*/*v*), which were incubated at 37 °C for 24h. After incubation, a loopful (0.1 mL) of the broth was then spread on Baird Parker agar (Conda Pronadisa, Madrid, Spain) supplemented with 5% egg yolk and tellurite (Conda Pronadisa, Madrid, Spain) and incubated for 24 to 48h at 37 °C. Based on the macroscopic characteristics of the colony (morphology and color), up to five presumptive *S*. *aureus* colonies per positive sample were selected and sub-cultured onto brain–heart infusion agar (BHIA) (Biokar, Beauvais, France). Thus, the identification of *S*. *aureus* isolates was performed using Gram staining and conventional biochemical tests, such as catalase, DNAse, and coagulase. To validate the results of all microbiological analyses, the reference strain *S*. *aureus* ATCC 25923 was used as a positive control. All isolates previously identified by biochemical tests were confirmed to be *S*. *aureus* using matrix-assisted laser desorption/ionization–time of flight (MALDI-TOF) mass spectrometry with Biotyper software RTC 4.0 (Bruker Daltonics, Bremen, Germany) according to the manufacturer’s instructions. Briefly, one colony from each overnight culture in a BHIA plate was picked with a Pasteur pipette and smeared onto a spot on a MALDI target plate. Two spots were reserved for each *S*. *aureus* isolate. After the inoculation of spots with the colony, 1 µL of 70% formic acid (Sigma-Aldrich, Gillingham, UK) was applied over the spots. Once the spots had dried at room temperature, 1 μL of CHCA (α-cyano-4-hydroxycinnamic acid) matrix solution was applied over the spots and allowed to dry. The generated protein mass spectra profiles were compared to the reference spectra stored in the database by the pattern-matching algorithm in the software. Based on the correlation between the two spectra, scores ranging from 0.00 to 3.00 were determined. The MALDI-TOF MS results were interpreted according to the manufacturer’s recommendations as follows: correct species identification (>2.0), correct genus identification (1.7–2.0), and unreliable result (<1.7). All identified *S*. *aureus* isolates were stored in BHI broth (Conda Pronadisa, Madrid, Spain) with glycerol (30% *v/v*) at −20 °C for molecular analysis.

### 2.4. Molecular Characterization of S. aureus

#### 2.4.1. DNA Extraction

DNA extraction from the overnight cultures of *S*. *aureus* isolates grown on milk plate count agar (Bio Rad, Marnes-la-Coquette, France) was performed using an InstaGene Kit (Bio Rad, France), according to the manufacturer’s recommendations. A Nanodrop 1000 spectrophotometer (Thermo Scientific, Wilmington, DE, USA) was used to measure the DNA concentrations.

#### 2.4.2. Identification of *S. aureus* Isolates by PCR Amplification of 23S rRNA Gene

A simplex PCR assay, specific to the 23S rRNA gene, was used as described by Straub et al. [25] for the identification of *S*. *aureus* isolates, using the species-specific primers staur4 (5′-ACGGAGTTACAAAGGACGAC-3′) and staur6 (5′-AGCTCAGCCTTAACGAGTAC-3′). The amplified PCR products were electrophoresed in 2% (*w*/*v*) agarose gel and visualized by ethidium bromide (1 µg/mL) staining under Gel Doc EQ apparatus (Bio-Rad, France). A 1 kb DNA ladder (Promega, Lyon, France) was used as a molecular weight standard. The reference strain *S*. *aureus* FRI 361 was used as a positive control.

#### 2.4.3. Detection of Virulence-Encoding Genes

Eleven staphylococcal enterotoxin genes were analyzed in all the *S*. *aureus* isolates obtained in this study according to the method described by Roussel et al. [26] and validated by the European reference laboratory for coagulase staphylococci (EURL CPS). For this, two multiplex PCRs were performed. The first multiplex PCR (mPCR1) was carried out to detect six enterotoxin genes, including *sea*, *seb*, *sec*, *sed*, *see*, and *ser*. In addition, the second multiplex PCR (mPCR2) was performed to detect five of the new enterotoxin genes (*seg*, *seh*, *sei*, *sej*, and *sep*). The amplified PCR products were electrophoresed in a 2% agarose gel and visualized using the Gel Doc EQ apparatus (Bio Rad, Marnes-la-Coquette, France). Five reference *S*. *aureus* strains (i.e., FRIS6, 374F, FRI137, FRI326, and FRI361) were used as positive controls. In parallel, a multiplex PCR was used to detect the presence of the genes encoding Panton–Valentine leukocidin (*luk*F/*luk*S-PV) and toxic shock syndrome toxin (*tst*) according to the protocol described by Benito et al. [27].

#### 2.4.4. Genetic Characterization of Isolated Strains

The obtained *S*. *aureus* isolates were typed by sequencing the repeat region of the *Staphylococcus* protein A gene (*spa*), previously obtained by PCR [8]. The obtained sequences were analyzed by Ridom Staph-Type software, through the detection and assignment of *spa* repeats (http://spaserver.ridom.de/, accessed on 10 July 2023). A specific PCR assay for the detection the *sau1*-*hsdS1* variant was performed to detect the presence of CC398 clones among our *S*. *aureus* isolates, as developed by Stegger et al. [28].

Multilocus sequence typing (MLST) was also performed in selected *S*. *aureus* isolates (one isolate of each *spa*-type). The 7 house-keeping genes (*arcC*, *aroE*, *glpF*, *gmk*, *pta*, *tpi*, and *yqiL*) of the *S*. *aureus* isolates were amplified as previously described [29], and the sequence types (STs) were assigned based on sequence analyses on the MLST database (http://pubmlst.org/, accessed on 17 July 2023). The clonal complexes (CCs) of the isolates were assigned according to their sequence types (STs); the same CCs were assigned to all isolates of the same *spa*-type.

### 2.5. Antimicrobial Susceptibility of S. aureus Isolates and Detection of mecA/mecC Genes

All *S*. *aureus* isolates were tested for their susceptibility to a panel of eight antibiotic molecules using the disc diffusion method and according to the guidelines of the Clinical and Laboratory Standards (CLSI) [30]. The antimicrobial agents tested included (µg/disk): penicillin G (10 UI), cefoxitin (30), gentamicin (10), tetracycline (30), erythromycin (15), ofloxacin (15), chloramphenicol (30), and trimethoprim/sulfamethoxazole (1.25/23.75). The strains were classified as susceptible, intermediate, or resistant according to the CLSI breakpoints [30]. The control strain *S*. *aureus* ATCC 25923 was used in susceptibility testing. Based on the obtained antimicrobial resistance phenotypes, a multiplex-PCR was performed as described by Stegger et al. [31] to confirm the MRSA strains through the detection of the *mecA* and/or *mecC* genes.

### 2.6. Biofilm Formation Ability In Vitro

#### 2.6.1. Congo Red Agar Method (CRA)

To determine their capacity to produce slime, all *S*. *aureus* isolates were cultivated on Congo red agar (CRA) plates containing brain–heart infusion broth (Conda Pronadisa, Spain) 37 g/L, sucrose (Biochem Chemopharma, Cosne-Cours-sur-Loire, France) 50 g/L, agar (Biokar, Beauvais, France) 10 g/L, and Congo red 0.8 g/L, as described by Freeman et al. [32]. The cultures were incubated aerobically for 24 to 48 h at 37 °C. After incubation, the colors of the colonies formed in this media were observed. Thus, isolates that showed black colonies were considered slime producers, while the non-slime producer isolates formed red colonies.

#### 2.6.2. Microtiter Plate Assay (MPA)

The quantitative MPA method described by Stepanović et al. [33], with some modifications, was used to assess the capacity of all *S*. *aureus* isolates to form biofilms. Briefly, *S*. *aureus* isolates from frozen stocks (−20 °C) were grown overnight at 37 °C in 5 mL brain–heart infusion broth (Conda Pronadisa, Madrid, Spain) and then cultivated in brain–heart infusion agar (BHIA) at 37 °C for 24 h under aerobic conditions. The next day, two to three colonies of each *S*. *aureus* culture were inoculated into 5ml of Trypticase Soy Broth (TSB) (Conda Pronadisa, Madrid, Spain) supplemented with 1% of Glucose (Sigma-Aldrich, Isère, France) and incubated overnight at 37 °C without shaking. The obtained cultures were then diluted in TSB-1% glucose. A quantity of 200 µL of diluted culture were transferred to three wells of a 96-well, flat-bottomed, tissue culture-treated plate (ProLab Scientific Co Ltd., Hangzhou, China). The reference strain *S*. *aureus* ATCC25923 was used as a positive control, and the medium TSB-1% glucose served as a negative control. After the overnight incubation of micro-plates at 37 °C, these were gently overturned onto paper towels to remove the liquid and non-adhered cells from the wells. Each well was gently washed three times with phosphate-buffered saline (PBS) and allowed to dry. Adherent bacteria were fixed with methanol (Honeywell, Seelze, Germany) for 15 mn. Finally, the biofilm formed was stained with 150 μL of 0.5% crystal violet (Biochem Chemopharma, Nièvre, France) for 15 min. After staining, the plates were rinsed with PBS. The adherent biofilm in each well was dissolved by the addition of 150 µL of ethanol (Honeywell, Seelze, Germany). Biofilm formation was assessed by measuring the optical density (OD) of each well at 560nm using a microtiter plate reader (Gentaur, Paris, France). The average OD value of all tested strains (ODs) and negative controls was calculated. The cut-off OD (ODc) is defined as three standard deviations above the mean OD of the negative control. In terms of biofilm production, considering the results of the microtiter plate test, the isolates were classified into the four following categories based on their optical density: non-biofilm producer (OD test < ODc), weak biofilm producer (ODc < OD < 2X ODc), moderate biofilm producer (2X ODc < OD < 4X ODc), and strong biofilm producer (4X ODc < OD).

## 3. Results

### 3.1. Prevalence of S. aureus

A total of 62 *S*. *aureus* isolates were obtained from 36 positive samples (one or two isolates per positive sample) out of 232 nasal swabs collected from various goat farms located in the Tizi Ouzou and Bouira areas (Algeria) (Table 1).

### 3.2. Detection of Virulence Factors in S. aureus Isolates

Regarding the virulence factors, 30 of the 62 *S*. *aureus* isolates (48.4%) harbored one or more staphylococcal enterotoxin genes. Overall, nine staphylococcal enterotoxin genes were detected among our isolates (*sea*, *seb*, *sec*, *sed*, *ser*, *sei*, *seg*, *sej*, and *sep*). Twelve staphylococcal genotypes related to enterotoxins were observed; the most frequently detected was *sec* (19.3%), followed by *sea* (9.7%). The other genotypic profiles were identified with lower frequencies (Table 2).

**Figure 2:**
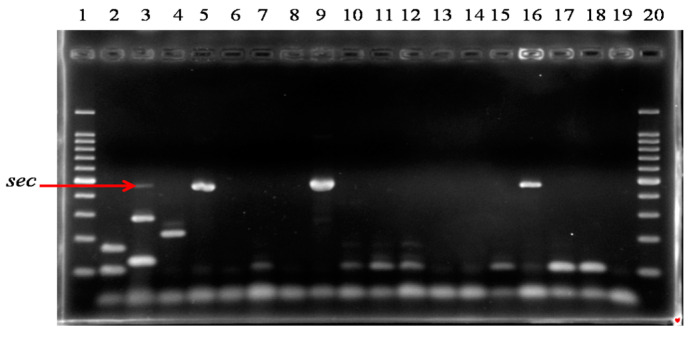
mPCR1 showing results of amplification products for staphylococcal enterotoxin genes after electrophoresis in Agarose gel. Lanes 1, 20: DNA molecular size markers (100pb); Lane 2: positive control for *sea* and *seb* (FRI S6); Lane 3: positive control for *sec*, *sed*, and *ser* (FRI361); Lane 4: positive control for *see* (FRI326); Lane 5: positive control for *sec* (FRI137); Lanes 6–18: tested isolates; Lane 19: negative control.

**Figure 3:**
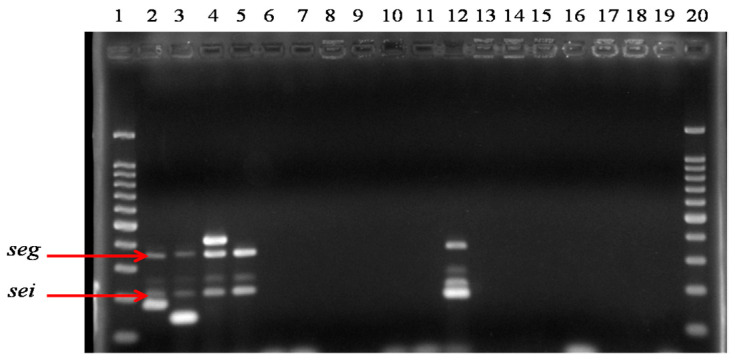
mPCR2 showing results of amplification products for staphylococcal enterotoxin genes after electrophoresis in Agarose gel. Lanes 1, 20: DNA molecular size markers (100pb); Lane 2: positive control for *sei*, *seg*, and *seh* (FRI 137); Lane 3: positive control for *seg*, *sei*, and *sej* (FRI361); Lane 4: positive control for *sep*, *sei*, and *seg* (FRI367); Lanes 5–18: tested isolates; Lane 19: negative control.

Figure 2 and Figure 3 show the PCR amplification products for staphylococcal enterotoxin genes in some of the isolates.

The gene *tst* encoding the toxic shock syndrome toxin was detected in fifteen isolates (24.2%). None of our isolates harbored the *pvl* gene encoding for the PVL toxin.

### 3.3. Molecular Characterization of S. aureus Isolates

*Spa*-typing was used to characterize the *S*. *aureus* isolates, and the results are shown in Table 3. Nine distinct *spa*-types were identified, with one being a novel type and registered in the database (t21230). The *spa*-type could not be determined for six of the isolates (non-typable). MLST was performed in one isolate of each *spa*-type, and it could be assigned to seven of these isolates, but it could not be performed in the last two minority *spa*-types (t2802 and t1534, with only one isolate each) (Table 3). All isolates of the same *spa*-type were assigned to the same ST/CC. All isolates were ascribed to four distinct STs, included in three clonal complexes: (1) ST5-ST6-CC5 (*spa*-types t21230, t11363, t688, t405, and t701) detected in 51.8% of isolates; (2) ST700-CC130/CC700 (*spa*-type t1773), detected in 42.6% of isolates; and (3) ST88-CC88 (*spa*-type t2649), detected in 5.5% of isolates (Table 3).

### 3.4. Antibiotic Susceptibility of S. aureus Isolates

The antibiotic susceptibility of the *S*. *aureus* isolates revealed that 34 of them (54.8%) were resistant to at least one antimicrobial agent. The highest resistance rate was found for penicillin G (51.6%). Lower resistance values were detected for other antimicrobial agents, as is the case for erythromycin (8%), tetracycline (4.8%), gentamicin (4.8%), chloramphenicol (4.8%), sulfamethoxazole/trimethoprim (3.2%), and ofloxacin (1.6%) (Table 4).

A multidrug-resistant phenotype was found in five *S. aureus* isolates (Table 5). We identified two MRSA isolates (cefoxitin-resistant) from the same goat on the same farm, and they harbored the *mecA* gene, representing low prevalence among the collected isolates (3.2%) and among the tested goats (0.4%) (Table 5).

The typing of the two MRSA isolates indicated that they belonged to the same sequence type (ST5) and clonal complex (CC5), but they were ascribed to two different *spa*-types: t450 and t688. The two MRSA isolates were multidrug-resistant and harbored enterotoxin genes, and one of them carried the *tst* gene.

### 3.5. Biofilm Formation Ability In Vitro

In total, 27 (43.5%) of the *S*. *aureus* isolates were slime producers (Table 6). Later, the isolates were assessed for confirmation of their ability to form biofilms in MPA, and it was observed that all *S*. *aureus* isolates obtained in this study could produce biofilms, among which 33 (53.2%) isolates had strong biofilm formation, 16 (25.8%) isolates were moderate biofilm producers, and the remaining isolates (21%) were weak biofilm producers (Table 6).

## 4. Discussion

*Staphylococcus aureus* is one of the main carriers of new and re-emerging antibiotic resistance determinants that represent a health risks for humans and animals [15]. It is a common commensal bacterium both in humans and animals. Livestock animals represent a major source for antimicrobial-resistant bacteria, the transmission of which can occur either through contact with colonized animals and/or through the consumption of their products, such as meat, milk, and eggs [13]. In this context, the aims of this study were to determine the prevalence of *S*. *aureus* in the nasal swabs of healthy dairy goats collected in various areas of Tizi Ouzou and Bouira (Algeria) and to investigate the phenotypic and genotypic characteristics of the isolated strains.

In this study, a low prevalence of *S*. *aureus* was observed among the nasal samples of healthy dairy goats, with different rates depending to the sampling regions. These results are in accordance with those of previous studies conducted in Algeria [34], Tunisia [35] and Saudi Arabia [36], with rate values of 11.9%, 10.2%, and 19.2%, respectively. However, higher frequencies were reported in Denmark [37], China [38], and Korea [39], with rates of 64%, 43.24%, and 82%, respectively. As is known, many factors could have an influence on the prevalence of *S*. *aureus*, including livestock density, isolation methods, breeding practices, and geographical conditions [15,40].

The results of this study showed that isolated *S*. *aureus* carried staphylococcal enterotoxin genes. Our results agree with those of other authors who have shown the presence of staphylococcal enterotoxin genes in *S*. *aureus* of goat origin [35,38,39]. In this study, the most frequent enterotoxin genes carried by the *S*. *aureus* isolates were *sec* and *sea*, which corroborate the results obtained by Gharsa et al. [35], who found a high prevalence of *sec* and *sea* among *S*. *aureus* isolates of goat origin. As reported by Normano et al. [41], staphylococcal enterotoxin C has been implicated in high number of staphylococcal food poisoning instances associated with the consumption of dairy products. Fifteen isolates harbored the *tst* gene encoding for the toxic shock syndrome toxin, which is consistent with findings among *S*. *aureus* of goat origin [35,42,43]. None of *S*. *aureus* isolates carried the Panton–Valentine toxin (*lukF/S*-PV) gene, an important virulence factor associated with pathogenicity. This contrasted with other studies, where the *pvl* gene was observed both in goat nasal carriages and goat milk [35,38,43,44]. As reported by Abdullahi et al. [15], *S*. *aureus* isolates of animal origin harbored several virulence factors, including *luk-S*/*F-PV*, *tst*, *eta*, *etb*, and the enterotoxin genes, which could have an impact on public health, mainly if these isolates are implicated in human or animal infections.

Among our *S. aureus* isolates, we found nine distinct *spa*-types and we also detected one new *spa*-type (t21230), suggesting that information about the population structure of *S*. *aureus* of goat origin is still limited, despite several studies having been conducted in this field [35,37,39,45]. Four STs were identified, including ST700, ST6, ST5, and ST88, which were assigned to three clonal lineages, including CC5, CC130/CC700, and CC88. CC5 was the most predominant in our study, including 28 isolates (51.8%). These results do not agree with those found by Shittu et al. [43] in Nigeria, Porrero et al. [45] in Spain, Saei and Panahi. [46] in Iran, and Gharsa et al. [35] in Tunisia, in which CC133 and CC522 were the predominant clones among goat populations. As reported by Aires-de-Sousa. [47], CC5 seems to be predominant among poultry, in which it is frequently implicated in disease. However, the host jumps lead to specific lineages spreading and adapting within new animal hosts [48]. ST700 associated with *spa*-type t1773 was the second most prevalent genetic lineage in our study (42.6%). As a single locus variant of ST130 (*tsi* allele different between them), the ST700 lineage is part of CC130 [49]; however, due to their distinct epidemiology and their independent evolution, ST700 and some of its single-locus variants may be considered a separate lineage (CC700) [50]; for this reason we included ST700 associated with both CCs. This ST700 lineage has previously been detected in ovine mastitis cases in Italy [51], nasal carriages in healthy goats and sheep in Tunisia [35,52], zoo animals in Germany [53], and abscesses of the submandibular lymph nodes of adult chamois in the Italian Alps [54]. The CC130 clonal complex has been associated in other studies with MRSA through the *mecC* mechanism in isolates of various hosts, including livestock, wildlife, companion animals, and humans, as well as environmental samples (wastewater and river water) [55]. Three isolates were assigned in our study to CC88 and were associated with the *spa*-type t2649. This lineage was also obtained from the nasal carriages of inpatients and hospital staff in Ghana [56].

In the present study, approximately half of the *S*. *aureus* isolates exhibited resistance to penicillin; although this rate is high, in general, it is lower than the values detected in human clinical isolates (>80%) [57]. Nevertheless, the rate detected in our study is in agreement with previous findings of other authors in animal isolates [34,36,43,46]. As reported by González-Candelas et al. [58], the use of antibiotics in human and veterinary medicine, agriculture farming, and other areas can promote the selection and emergence of antibiotic-resistant organisms. The collection of *S. aureus* isolates showed low resistance rates to tetracycline, erythromycin, gentamicin, sulfamethoxazole/trimethoprim, chloramphenicol, and ofloxacin. The same results were obtained in previous studies [34,35,43,46]. The use of phenicols in the veterinary sector (as in the case of florfenicol) may promote the emergence of resistance to chloramphenicol; this group of antibiotics (phenicols) could coselect for resistance to different classes of antibiotics (including linezolid) [15].

It is necessary to conduct routine surveillance on MRSA clones of animal origin to gain a better understanding of the transmission routes of new lineages and for implementing appropriate preventive and control measures. Only two isolates (3.2%) were identified as MRSA, representing only 0.4% of the goats tested. A low prevalence was observed in other studies conducted in Saudi Arabia [36], Spain [59], Korea [39], and Nigeria [43], with values of 0.8%, 15.8%, 1.2%, and 4.4%, respectively. The detection of MRSA among our *S*. *aureus* isolates highlights the public health risks associated with the consumption of contaminated milk and the spread of potential zoonotic lineages between animals and humans, even though the prevalence of MRSA in our study was low. Published data report the zoonotic transmission of *S*. *aureus* between livestock and humans, especially people who work with farm animals [60,61]. Moreover, veterinarians and veterinary students were the most exposed to certain staphylococci predominantly found in farm animals [23]. Their transmission may occur through direct contact with colonized animals and through the handling and consumption of contaminated food of animal origin [13]. In our study, the two MRSA isolates were resistant to antimicrobial agents other than β-lactams, including tetracycline, macrolides (erythromycin), aminoglycosides (gentamicin), and chloramphenicol, indicating a multidrug-resistant phenotype, as in other studies [36,39,43,59]. None of the MRSA isolates harbored genes encoding Panton–Valentine leucocidin (*lukF*/*S*-PV), although these genes have been reported in the nasal carriages of goats [43]. Similar to our results, previous studies have reported the ability of MRSA isolates of goat origin to carry staphylococcal enterotoxin genes [39]. With regard to genetic typing, the two MRSA isolates recovered in this study belonged to the same CC (CC5) and ST (ST5), but they were ascribed to two different *spa*-types: t450 and t688. Our results are in accordance with those of Titouche et al. [62], who isolated *spa*-types t450 and t688 (ST5) from raw and acidified milk (rayeb), respectively. Since ST5 has been observed in humans as well as in many domesticated animals, it can currently be considered an animal-adapted clone [63]. However, the globalization of the broiler poultry sector was subsequently responsible for the dissemination of *S*. *aureus* CC5 [64].

Bacterial cells have a tendency to adhere to solid surfaces and accumulate in multi-layered cell clusters called biofilms, with their microbial physiology being distinct from the planktonic state [65]. This also applies to *S*. *aureus*, which has the ability to form a biofilm, as part of its normal life cycle [66]. Their capacity to form a biofilm allows microorganisms to survive in hostile environments and to resist conventional treatments [67]. However, few data were available concerning the biofilm formation ability of isolates of animal origin, and most of them were focused on bovine mastitis [68]. As reported by Pedersen et al. [69], the role of biofilms in bovine mastitis is still unclear, and more in vivo studies are required to gain a better understanding of the actual role of biofilm formation in the pathogenesis of bovine mastitis. In this study, we used two techniques to evaluate the capacity of recovered isolates to produce biofilms in vitro. Among all the recovered isolates, 27 (43.5%) were found to be biofilm producers using the CRA method. Our results show a greater difference with those of Lira et al. [70], who reported a rate of 28% in a CRA test. Although the CRA test is not considered the most sensitive for determining biofilm formation, this simple qualitative phenotypic test is used for its acceptable sensitivity and specificity [71,72]. However, multiple factors, such as glucose and sodium chloride, among others, affect the slime production of *Staphylococcus* spp. [73]. The MPA test revealed that all isolates showed an ability to produce biofilms, which is similar to the results obtained by Silva et al. [68] in *S*. *aureus* isolates from different animal species, including pets, livestock, and wild animals. Biofilms that are produced on food contact surfaces in the food industry are of great interest in food hygiene because they can harbor pathogenic and spoilage bacteria and cause contamination during post-processing, leading to a decrease in the shelf life of products and the transmissions of diseases [74].

## 5. Conclusions

This study showed that the nares of healthy goats could be a reservoir of toxigenic and multidrug-resistant *S*. *aureus*. Clonal diversity in *S*. *aureus* isolates was observed, with a predominance of CC5. The presence of CC130/CC700 among our MSSA isolates is interesting, since the CC130 lineage is associated with *mecC* in the MRSA variant from human and animal isolates in Europe. The evolution of the CC130 lineage in both MSSA and MRSA of different niches is a subject of interest. Further expanded studies covering an extensive *S*. *aureus* population from different animal species collected in various geographical locations would give more information about the genetic lineages colonizing and infecting different livestock animals and their dissemination in the country.

## Figures and Tables

**Table 1:** Number and distribution of goat nasal samples carrying *S*. *aureus* isolates from animals on goat farms in four regions of two provinces of Algeria.

Provinces	Regions	Number of Herds	Number of Collected Samples	Number and % of Samples Carrying *S. aureus* Isolates
Tizi Ouzou	Benni Yenni	1	36	3 (8.3)
	Ain El Hammam	1	15	2 (13.3)
	Azeffoun	9	168	29 (17.3)
Bouira	Sor El Ghozlane	2	13	2 (15.4)
Total		13	232	36 (15.5)

**Table 2:** Distribution of enterotoxin gene profiles among the 62 *S*. *aureus* isolates of goat origin.

Enterotoxin Gene Profile	Number and % of *S*. *aureus* Isolates
*sea*	6 (9.7)
*sec*	12 (19.3)
*sep*	1 (1.6)
*sea* + *ser*	1 (1.6)
*sea* + *sec*	1 (1.6)
*sea* + *sei*	1 (1.6)
*sec* + *ser*	1 (1.6)
*sea* + *seb* + *ser*	2 (3.2)
*sea* + *seb* + *sec* + *ser*	1 (1.6)
*sec* + *sed* + *ser* + *sej*	1 (1.6)
*sed* + *ser* + *seg* + *sei* + *sej*	2 (3.2)
*sea + seb* + *serd*+ *ser* + *sej* + *sep*	1 (1.6)
Total	30 (48.4%)

**Table 3:** Phenotypic and genotypic characteristics of the 62 *S*. *aureus* isolates recovered from nasal swabs of dairy goats in this study.

*Spa*-Type	ST/CC	Number of Isolates	Area of Farm ^a^	Virulence Genes Detected ^b^	Phenotype of Resistance ^b,c^	*mecA*/*mecC* Genes
t1773	ST700/CC130-CC700	23	F1, F3, F4, F5, F7, F9, F10, F13	*tst*^(9)^, *sea*^(5)^, *seb*^(2)^, *sec*^(12)^, *sed*^(1)^, *ser*^(4)^, *sei*^(1)^, *sej*^(1)^	PEN^(11)^, TET^(1)^, ERY^(1)^, OFL^(1)^	
t11363	ST6/CC5	15	F3, F4, F5, F6, F9	*tst*^(1)^, *sea*^(4)^, *seb*^(1)^, *ser*^(1)^	PEN^(9)^, ERY^(1)^, SXT^(1)^	
t701	ST5/CC5	7	F7	*tst*^(2)^, *sea*^(2)^	PEN^(4)^, GEN^(1)^	
t21230	ST6/CC5	4	F12, F13	*tst*^(1)^, *sec*^(1)^		
t2802	NT ^d^	1	F7		PEN^(1)^, ERY^(1)^, SXT^(1)^	
t450	ST5/CC5	1	F7	*sed*^(1)^, *ser*^(1)^, *seg*^(1)^, *sei^(^*^1)^, *sej*^(1)^	PEN^(1)^, CEF^(1)^, TET^(1)^, ERY^(1)^, GEN^(1)^, CHL^(1)^	*mecA*
t688	ST5/CC5	1	F7	*tst*^(1)^, *sed*^(1)^, *ser*^(1)^, *seg*^(1)^, *sei^(^*^1)^, *sej*^(1)^	PEN^(1)^, CEF^(1)^, TET^(1)^, ERY^(1)^, CHL^(1)^	*mecA*
t1534	NT ^d^	1	F9	*sea*^(1)^, *sec*^(1)^		
t2649	ST88/CC88	3	F13	*tst*^(1)^*, sea*^(1)^, *seb*^(1)^, *sec*^(1)^, *sed*^(1)^, *ser*^(1)^, *sej*^(1)^, *sep*^(2)^	PEN^(3)^	
Non-typable	Non-typable	6	F2, F3, F5, F7, F9, F12	*sec*^(2)^, *ser*^(1)^	PEN^(3)^, CHL^(1)^, GEN^(1)^	

^a^ Geographical areas of the farms: F1, F2: Sor El Ghozlane (Bouira); F3: Beni Yenni (Tizi Ouzou); F4: Ain El Hammam (Tizi Ouzou); F5, 6, 7, 9, 10, 12, 13: Azeffoun (Tizi Ouzou); ^b^ in the superscript, we have indicated the number of isolates with this characteristic; ^c^ PEN: penicillin; CEF: cefoxitin; TET: tetracycline; ERY: erythromycin; GEN: gentamicin; CHL: chloramphenicol; SXT: sulfamethoxazole/trimethoprim; ^d^ NT: not tested.

**Table 4:** Antibiotic resistance rates of the collection of 62 *S*. *aureus* isolates from goat nasal samples.

Antibiotics	No (%) of *S*. *aureus*		
	Resistant	Intermediate	Susceptible
Penicillin G	32 (51.6)	0 (0)	30(48.4)
Cefoxitin	2 (3.2)	0 (0)	60 (96.8)
Chloramphenicol	3 (4.8)	0 (0)	59 (95.2)
Erythromycin	5 (8)	3 (3.2)	54 (87.1)
Gentamicin	3 (4.8)	0 (0)	59 (95.2)
Tetracycline	3 (4.8)	10 (16.1)	49 (79)
Sulfamethoxazole/trimethoprim	2 (3.2)	0 (0)	60 (96.8)
Ofloxacin	1 (1.6)	0 (0)	61 (98.4)

**Table 5:** Multidrug resistance profiles observed in *S. aureus* isolates from nasal swabs of healthy goats.

Antimicrobial MDR Resistance Phenotype ^1^	No. of Isolates with Phenotype (% with Respect to *S*. *aureus*)	*mecA* Gene
PEN-CEF-TET-ERY-GEN-CHL	1 (1.6)	+
PEN-CEF-TET-ERY-CHL	1 (1.6)	+
PEN-ERY-SXT	2 (3.2)	−
PEN-ERY-OFL	1(1.6)	−
Total	5 (8.1)	

^1^ PEN = penicillin G; CEF = cefoxitin; TET = tetracycline; ERY = erythromycin; GEN = gentamicin; CHL = chloramphenicol; SXT = sulfamethoxazole/trimethoprim; OFL = ofloxacin.

**Table 6:** Distribution of slime and biofilm-producing *S*. *aureus* isolates recovered from nasal carriage of healthy goats (*n* = 62).

Criteria	Number and % of Isolates		
Slime-producing (CRA performance)	Positive		27 (43.5)
	Negative		35 (56.4)
Biofilm-producing (MPA performance)	Positive	Weak formation	13 (21)
		Moderate formation	16 (25.8)
		Strong formation	33 (53.2)
		Total	62 (100)
	Negative		0 (0)

CRA: Congo red agar; MPA: microtiter plate assay.

## Data Availability

The data are included in the article/referenced in article.

## References

[1] Pokharel S., Shrestha P., Adhikari B. (2020). Antimicrobial use in food animals and human health: Time to implement One Health approach. Antimicrob. Resist. Infect. Control.

[2] WHOA (2022). Annual Report on Antimicrobial Agents Intended for Use in Animals.

[3] Tollefson L., Karp B.E. (2004). Human health impact from antimicrobial use in food animals. Med. Mal. Infect..

[4] Van Boeckel T.P., Pires J., Silvester R., Zhao C., Song J., Criscuolo N.G., Gilbert M., Bonhoeffer S., Laxminarayan R. (2019). Global trends in antimicrobial resistance in animals in low-and middle-income countries. Science.

[5] Rodrigues I.d.A., Ferrari R.G., Panzenhagen P.H.N., Mano S.B., Conte-Junior C.A. (2020). Antimicrobial resistance genes in bacteria from animal-based foods. Adv. Appl. Microbiol..

[6] Despotovic M., de Nies L., Busi S.B., Wilmes P. (2023). Reservoirs of antimicrobial resistance in the context of One Health. Curr. Opin. Microbiol..

[7] Martínez-Álvarez S., Sanz S., Olarte C., Hidalgo-Sanz R., Carvalho I., Fernández-Fernández R., Campaña-Burguet A., Latorre-Fernández J., Zarazaga M., Torres C. (2022). Antimicrobial Resistance in *Escherichia coli* from the Broiler Farm Environment, with Detection of SHV-12-Producing Isolates. Antibiotics.

[8] Abdullahi I.N., Lozano C., Simon C., Latorre-Fernandez J., Zarazaga M., Torres C. (2023). Nasal staphylococci community of healthy pigs and pig-farmers in Aragon (Spain). Predominance and within-host resistome diversity in MRSA-CC398 and MSSA-CC9 lineages. One Health.

[9] Verkade E., Kluytmans J. (2004). Livestock-associated *Staphylococcus aureus* CC398: Animal reservoirs and human infections. Infect. Genet. Evol..

[10] Lowy F.D. (1998). *Staphylococcus aureus* infections. N. Engl. J. Med..

[11] Fitzgerald J.R. (2012). Livestock-associated *Staphylococcus aureus*: Origin, evolution and public health threat. Trends Microbiol..

[12] Peton V., Le Loir Y. (2014). *Staphylococcus aureus* in veterinary medicine. Infect. Genet. Evol..

[13] Crespo-Piazuelo D., Lawlor P.G. (2021). Livestock-associated methicillin-resistant *Staphylococcus aureus* (LA-MRSA) prevalence in human in close contact with animals and measures to reduce on-farm colonization. Ir. Vet. J..

[14] Cuny C., Köck R., Witte W. (2013). Livestock-associated MRSA (LA-MRSA) and its relevance for humans in Germany. Int. J. Med. Microbiol..

[15] Abdullahi I.N., Lozano C., Saidenberg A.B.S., Latorre-Fernández J., Zarazaga M., Torres C. (2023). Comparative review of the nasal carriage and genetic characteristics of *Staphylococcus aureus* in healthy livestock: Insight into zoonotic and anthroponotic clones. Infect. Genet. Evol..

[16] Hartman B.J., Tomasz A. (1984). Low-affinity penicillin-binding protein associated with beta-lactam resistance in *Staphylococcus aureus*. J. Bacteriol..

[17] Schmidt T., Kock M.M., Ehlers M.M. (2015). Diversity and antimicrobial susceptibility profiling of staphylococci isolated from bovine mastitis cases and close human contacts. J. Dairy Sci..

[18] Lakhundi S., Zhang K. (2018). Methicillin-resistant *Staphylococcus aureus*: Molecular characterization, evolution, and epidemiology. Clin. Microbiol. Rev..

[19] Graveland H., Duim B., van Duijkeren E., Heederik D., Wagenaar J.A. (2011). Livestock-associated methicillin-resistant *Staphylococcus aureus* in animals and humans. Int. J. Med. Microbiol..

[20] Catry B., Van Duijkeren E., Pomba M.C., Greko C., Moreno M.A., Pyörälä S., Ruzauskas M., Sanders P., Threlfall E.J., Ungemach F. (2010). Reflection paper on MRSA in food-producing and companion animals: Epidemiology and control options for human and animal health. Epidemiol. Infect..

[21] Spoor L.E., McAdam P.R., Weinert L.A., Rambaut A., Hasman H., Aarestrup F.M., Kearns A.M., Larsen A.R., Skov R.L., Fitzgerald J.R. (2013). Livestock origin for a human pandemic clone of community-associated methicillin-resistant *Staphylococcus aureus*. mBio.

[22] Cuny C., Wieler L.H., Witte W. (2015). Livestock-associated MRSA: The impact on Humans. Antibiotics.

[23] Abdullahi I.N., Lozano C., Ruiz-Ripa L., Fernández-Fernández R., Zarazaga M., Torres C. (2021). Ecology and genetic lineages of nasal *Staphylococcus aureus* and MRSA carriage in healthy persons with or without animal-related occupational risks of colonization: A review of global reports. Pathogens.

[24] Silva V., Correira S., Pereira J.E., Igrejas G., Poeta P., Hashmi M.Z. (2020). Surveillance and environmental risk assessment of antibiotics and AMR/ARGs related with MRSA: One health perspective. Antibiotics and Antimicrobial Resistance Genes: Environmental Occurrence and Treatment Technologies.

[25] Straub J., Hertel C., Hammes W.P. (1999). A 23S rDNAtargeted polymerase chain reaction-based system for detection of *Staphylococcus aureus* in meat starter cultures and dairy products. J. Food Prot..

[26] Roussel S., Felix B., Vingadassalon N., Grout J., Hennekinne J.A., Guillier L., Brisabois A., Auvray F. (2015). *Staphylococcus aureus* strains associated with food poisoning outbreaks in France: Comparison of different molecular typing methods, including MLVA. Front. Microbiol..

[27] Benito D., Gomez P., Lozano C., Estepa V., Gómez-Sanz E., Zarazaga M., Torres C. (2014). Genetic lineages, antimicrobial resistance, and virulence in *Staphylococcus aureus* of meat samples in Spain: Analysis of immune evasion cluster (IEC) genes. Foodborne Pathog. Dis..

[28] Stegger M., Lindsay J.A., Moodley A., Skov R., Broens E.M., Guardabassi L. (2011). Rapid PCR detection of *Staphylococcus aureus* clonal complex 398 by targeting the restriction-modification system carrying sau1-hsdS1. J. Clin. Microbiol..

[29] Lozano C., Porres-Osante N., Crettaz J., Torres C., Porres-Osante N., Rojo-Bezares B., Sáenz Y., Torres C., Crettaz J., Olarte I. (2012). Changes in genetic lineages, resistance, and virulence in clinical methicillin-resistant *Staphylococcus aureus* in a Spanish hospitals. J. Infect. Chemother..

[30] CLSI (2020). Performance Standards for Antimicrobial Susceptibility Testing.

[31] Stegger M., Andersen P.S., Kearns A., Pichon B., Holmes M.A., Edwards G., Laurent F., Teale C., Skov R., Larsen A.R. (2012). Rapid detection, differentiation and typing of methicillin-resistant *Staphylococcus aureus* harbouring either *mecA* or the new *mecA* homologue *mecA* (LGA_251_). Clin. Microbiol. Infect..

[32] Freeman D.J., Falkiner F.R., Keane C.T. (1989). New method for detecting slime production by coagulase negative staphylococci. J. Clin. Pathol..

[33] Stepanović S., Vuković D., Hola V., Di Bonaventura G., Djukić S., Cirković I., Ruzicka F. (2007). Quantification of biofilm in microtiter plates: Overview of testing conditions and practical recommendations for assessment of biofilm production by staphylococci. APMIS.

[34] Mairi A., Touati A., Pantel A., Zenati K., Martinez A.Y., Dunyach-Remy C., Sotto A., Lavigne J.P. (2019). Distribution of toxinogenic methicillin-resistant and methicillin-susceptible *Staphylococcus aureus* from different ecological niches in Algeria. Toxins.

[35] Gharsa H., Ben Slama K., Gomez-Sanz E., Lozano C., Zarazaga M., Messadi L., Boudabous A., Torres C. (2015). Molecular characterization of *Staphylococcus aureus* from nasal samples of healthy farm animals and pets in Tunisia. Vector-Borne Zoonotic Dis..

[36] El-Deeb W., Fayez M., Elmoslemany A., Kandeel M., Zidan K. (2018). Methicillin-resistant *Staphylococcus aureus* among goat farms in Eastern province, Saudi Arabia: Prevalence and risk factors. Prev. Vet. Med..

[37] Eriksson J., Espinosa-Gongora C.E., Stamphøj I., Larsen A.R., Guardabassi L. (2013). Carriage frequency, diversity and methicillin-resistance of *Staphylococcus aureus* in Danish small ruminants. Vet. Microbiol..

[38] Zhou Z., Zhang M., Li H., Yang H., Li X., Song X., Wang Z. (2017). Prevalence and molecular characterization of *Staphylococcus aureus* isolated from goats in Chongping, China. BMC Vet. Res..

[39] Mechesso A.F., Moon D.C., Ryoo G.S., Song H.J., Chung H.Y., Kim S.U., Choi J.H., Kim S.J., Kang H.Y., Na S.H. (2021). Resistance profiling and molecular characterization of *Staphylococcus aureus* isolated from goats in Korea. Int. J. Food Microbiol..

[40] El-Ashker M., Monecke S., Gwida M., Saad T., El-Gohary A., Mohamed A., Reißig A., Frankenfeld K., Gary D., Müller E. (2022). Molecular characterization of methicillin-resistant and methicillin-susceptible *Staphylococcus aureus* clones isolated from healthy dairy animals and their caretakers in Egypt. Vet. Microbiol..

[41] Normano G., Corrente M., La Salandra G., Dambrosio A., Quaglia N.C., Parisi A., Greco G., Bellacicco A.L., Virgilio S., Celano G.V. (2007). Methicillin-resistant *Staphylococcus aureus* (MRSA) in foods of animal origin product in Italy. Int. J. Food. Microbiol..

[42] Merz A., Stephan R., Johler S. (2016). *Staphylococcus aureus* from goat and sheep milk seem to be closely related and differ from isolates detected from bovine milk. Front. Microbiol..

[43] Shittu A.O., Taiwo F.F., Froböse N.J., Schwartbeck B., Niemann S., Mellmann A., Schaumburg F. (2021). Genomic analysis of *Staphylococcus aureus* from the West African Swarf (WAD) goat in Nigeria. Antimicrob. Resist. Infect. Control.

[44] Mama O.M., Gomez-Sanz E., Ruiz-Ripa L., Gómez P., Torres C. (2019). Diversity of staphylococcal species in food producing animals in Spain, with detection of PVL-positive MRSA ST8 (USA300). Vet. Microbiol..

[45] Porrero M.C., Hasman H., Vela A.I., Fernández-Garayzábal J.F., Domínguez L., Aarestrup F.M. (2012). Clonal diversity of *Staphylococcus aureus* originating from the small ruminants goats and sheep. Vet. Microbiol..

[46] Saei H.D., Panahi M. (2020). Genotyping and antimicrobial resistance of *Staphylococcus aureus* isolates from dairy ruminants: Differences in the distribution of clonal types between cattle and small ruminants. Arch. Microbiol..

[47] Aires-de-Sousa M. (2017). Methicillin-resistant *Staphylococcus aureus* among animals: Current overview. Clin. Microbiol. Infect..

[48] Haag A.F., Fitzgerald J.R., Penadés J.R. (2019). *Staphylococcus aureus* in animals. Microbiol. Spectr..

[49] Gómez P., Ruiz-Ripa L., Fernández-Fernández R., Gharsa H., Ben Slama K., Höfle U., Zarazaga M., Holmes M.A., Torres C. (2021). Genomic analysis of *Staphylococcus aureus* of the lineage CC130, including *mecC*-carrying MRSA and MSSA isolates recovered of animal, Human, and environmental origins. Front. Microbiol..

[50] Smith E.M., Needs P.F., Manley G., Green L.E. (2014). Global distribution and diversity of ovine-associated *Staphylococcus aureus*. Infect. Genet. Evol..

[51] Azara E., Piras M.G., Parisi A., Tola S. (2017). Antimicrobial susceptibility and genotyping of *Staphylococcus aureus* isolates collected between 1986 and 2015 from ovine mastitis. Vet. Microbiol..

[52] Gharsa H., Ben Slama K., Lozano C., Gómez-Sanz E., Klibi N., Ben Sallem R., Gómez M., Zarazaga P., Boudabous A., Torres C. (2012). Prevalence, antibiotic resistance, virulence traits and genetic lineages of *Staphylococcus aureus* in healthy sheep in Tunisia. Vet. Microbiol..

[53] Feßler A.T., Thomas P., Mühldorfer K., Grobbel M., Brombach J., Eichhorn I., Monecke S., Ehricht R., Schwarz S. (2018). Phenotypic and genotypic characteristics of *Staphylococcus aureus* isolates from zoo and wild animals. Vet. Microbiol..

[54] Luzzago C., Locatelli C., Franco A., Scaccabarozzi L., Gualdi V., Viganò R., Sironi G., Besozzi M., Castiglioni B., Lanfranchi P. (2014). Clonal diversity, virulence-associated genes and antimicrobial resistance profile of *Staphylococcus aureus* isolates from nasal cavities and soft tissue infections in wild ruminants in Italian Alps. Vet. Microbiol..

[55] Zarazaga M., Gomez P., Ceballos S., Torres C., Fetsch A. (2018). Molecular epidemiology of *Staphylococcus aureus* lineages in the animal-human interface. Staphylococcus aureus.

[56] Egyir B., Guardabassi L., Nielsen S.S., Larsen J., Addo K.K., Newman M.J., Larsen A.R. (2013). Prevalence of nasal carriage and diversity of Staphylococcus aureus among inpatients and hospital staff at Korle Bu teaching hospital, Ghana. J. Glob. Antimicrob. Resist..

[57] Mama O.M., Aspiroz C., Lozano C., Ruiz-Ripa L., Azcona J.M., Seral C., Cercenado E., López-Cerero L., Palacian P., Belles-Belles A. (2021). Penicillin susceptibility among invasive MSSA infections: A multicentre study in 16 Spanish hospitals. J. Antimicrob. Chemother..

[58] González-Candelas F., Comas I., Martínez J.L., Galán J.C., Baquero F., Tibayrenc M. (2017). The evolution of antibiotic resistance. Genetics and Evolution of Infectious Diseases.

[59] Abreu R., Rodríguez-Álvarez C., Lecuona M., Castro-Hernández B., González J.C., Aguirre-Jaime A., Arias Á. (2019). Prevalence and characteristics of methicillin-resistant staphylococci in goats on the island of Tenerife, Spain. Acta. Vet. Hung..

[60] Locatelli C., Cremonesi P., Caprioli A., Carfora V., Ianzano A., Barberio A., Morandi S., Casula A., Castiglioni B., Bronzo V. (2017). Occurrence of methicillin-resistant *Staphylococcus aureus* in dairy cattle herds, related swine farms, and humans in contact with herds. J. Dairy Sci..

[61] Krukowski H., Bakula Z., Iskra M., Olender A., Bis-Wencel H., Jagielski T. (2020). The first outbreak of methicillin-resistant *Staphylococcus aureus* in dairy cattle in Poland with evidence of on-farm and intrahousehold transmission. J. Dairy Sci..

[62] Titouche Y., Houali K., Ruiz-Ripa L., Vingadassalon N., Nia Y., Fatihi A., Cauquil A., Bouchez P., Bouhier L., Torres C. (2020). Enterotoxin genes and antimicrobial resistance of *Staphylococcus aureus* isolated from food products in Algeria. J. Appl. Microbiol..

[63] Pantosti A. (2012). Methicillin-resistant *Staphylococcus aureus* associated with animals and its relevance to Human health. Front. Microbiol..

[64] Larsen A.R., Fitzgerald J.R., Larsen J., Sing A. (2023). Methicillin-resistant *Staphylococcus aureus* in food animals: Host-adaptive evolution, epidemiology, and public health threat. Zoonoses: Infections Affecting Humans and Animals.

[65] Azara E., Longheu C., Sanna G., Tola S. (2017). Biofilm formation and virulence factor analysis of *Staphylococcus aureus* isolates collected from ovine mastitis. J. Appl. Microbiol..

[66] Sivaraman G.K., Muneeb K.H., Sudha S., Shome B., Cole J., Holmes M. (2021). Prevalence of virulent and biofilm forming ST88-IV-t2526 methicillin-resistant *Staphylococcus aureus* clones circulating in local retail fish markets in Assam, India. Food Control.

[67] García A.B., Percival S.L., Percival S., Knottenbelt D., Cochrane C. (2011). Zoonotic infections: The role of biofilms. Biofilms and Veterinary Medicine.

[68] Silva V., Correia E., Pereira J.E., González-Machado C., Capita R., Alonso-Calleja C., Igrejas G., Poeta P. (2022). Biofilm formation of *Staphylococcus aureus* from pets, livestock, and wild animals: Relationship with clonal lineages and antimicrobial resistance. Antibiotics.

[69] Pedersen R.R., Krömker V., Bjarnsholt T., Dahl-Pedersen K., Buhl R., Jørgensen E. (2021). Biofilm research in bovine mastitis. Front. Vet. Sci..

[70] Lira M.C., Givisiez P.E.N., de Sousa F.G.C., Magnani M., De Souza E.L., Spricigo D.A., Gebreyes W.A., De Oliveira C.J.B. (2016). Biofilm-forming and antimicrobial resistance traits of staphylococci isolated from goat dairy plants. J. Infect. Dev. Ctries..

[71] Jain A., Agarwal A. (2009). Biofilm production, a marker of pathogenic potential of colonizing and commensal staphylococci. J. Microbiol. Methods.

[72] Ballah F.M., Islam S., Rana L., Ferdous F.B., Ahmed R., Pramanik P.K., Karmoker J., Ievy S., Sobur A., Siddique M.P. (2022). Phenotypic and Genotypic detection of biofilm-forming *Staphylococcus aureus* from different food sources in Bangladesh. Biology.

[73] Lee J.S., Bae Y.M., Han A., Lee S.Y. (2016). Development of congo red broth method for the detection of biofilm-forming or slime-producing *Staphylococcus* sp. LWT.

[74] Giaouris E.E., Simões M.V., Holban A.M., Mihai A. (2018). Pathogenic biofilm formation in the food industry and alternative control strategies. Food Diseases.

